# A survey of how frequently UK-based dermatology clinicians apply sun protection factor and the variables influencing this

**DOI:** 10.1093/skinhd/vzaf031

**Published:** 2025-04-29

**Authors:** Amber Blood, Áine Kelly, Emma Craythorne

**Affiliations:** School of Medicine, University of St Andrews, St Andrews, UK; St John's Institute of Dermatology, Guy’s and St Thomas’ Hospital, London, UK; St John's Institute of Dermatology, Guy’s and St Thomas’ Hospital, London, UK

## Abstract

This study evaluates sunscreen application practices among UK dermatology clinicians, revealing that only 10% meet recommended reapplication guidelines. Factors such as skin type, gender, product preferences and topical treatments significantly influence sun protection factor (SPF) use. Findings underscore the need for clearer clinical guidelines and practical solutions to enhance adherence and inform patient education.

Dear Editor, Doctors’ personal behaviours can influence the effectiveness of their preventive counselling.^1^ In dermatology, clinicians’ sun protection practices likely impact the frequency of sun safety discussions and their ability to address patient adherence challenges.

This study evaluates whether dermatologists’ sunscreen use aligns with guidelines and examines factors influencing their sun protection factor (SPF) application frequency, to inform patient education and public health strategies.

The personal use of SPF among dermatologists has not been studied. Limited research indicates that medical students apply SPF less frequently than recommended due to societal pressures,^2^ and only 8% of primary care physicians wear daily sun protection.^3^

The Skin Cancer Foundation^4^ advises applying SPF multiple times daily, year-round. While National Institute for Health and Care Excellence (NICE)^5^ guidelines do not explicitly recommend year-round sunscreen use, they highlight the risk of sunburn year-round. Despite past concerns that SPF use may reduce vitamin D levels,^6^ this has been disproven.^7^ Furthermore, NICE^5^ confirms that sunlight alone is insufficient for adequate vitamin D synthesis in the UK from October to March, supporting year-round sunscreen use alongside vitamin D supplementation.

Cultural and practical barriers also impact sun protection practices. Song *et al.*^8^ highlight the need for culturally competent care, as individuals with darker skin tones often struggle to find nonresidue sunscreens. Additionally, SPF-containing cosmetics can mislead users into believing one application provides all-day protection, underscoring the importance of reapplication.^9^

Dermatologists frequently discuss sun protection, and their recommendations are associated with higher sunscreen use. However, adherence in the general population remains low, emphasizing the need for better communication strategies.

This study aims to investigate the frequency of sunscreen application among dermatology clinicians in the UK. It also examines associations between application frequency and various factors, including clinician training grade, age, sex, Fitzpatrick skin type, pre-existing facial skin disease, sunscreen vehicle, brand preference and concurrent use of topical treatments. This study sought to obtain a comprehensive understanding of the determinants influencing sunscreen application practices in dermatology professionals.

A cross-sectional survey was electronically distributed to 400 members of the British Society for Dermatological Surgery and the British Cosmetic Dermatology Group on two occasions between 1 May and 31 July 2024. The survey assessed participants’ daily sunscreen use. Data were analysed using Microsoft Excel.

A total of 154 responses were received from 91 consultant dermatologists, 28 registrars and 35 general practitioners (GPs) with an extended role (GPwER) in dermatology. Responses from specialty and associate specialist doctors, nurses and GPs (fewer than two responses each) were excluded. This provided 99.9% power to detect a moderate effect size (0.5) at a significance level of 0.05.

GPwERs were the most likely to apply SPF more than once daily (14%, *n* = 5/35), followed by consultants (10%, *n* = 9/91) and registrars (7%, *n* = 2/28) (Table 1). Women were twice as likely as men to apply SPF more than once daily (12% vs. 6%).

**Table 1:** Sun protection by variable

Relationship between a variable and SPF application frequency			SPF application frequency							
Independent variable	No. of responses	% of responses	More than once daily	% of variable	Once daily	% of variable	Only when sun-exposed	% of variable	Does not use	% of variable
Sun protection by training grade: χ^2^ (6, *N* = 154) = 16, *P* = 0.01										
Consultant	91	59	9	10	34	37	43	47	5	6
Registrar	28	18	2	7	20	71	5	18	1	4
GPwER	35	23	5	14	22	63	7	20	1	3
Sun protection by sex: χ^2^ (3, *N* = 154) = 19, *P* < 0.001										
Male	47	31	3	6	15	32	23	49	6	13
Female	107	69	13	12	61	57	32	30	1	1
Sun protection by age: χ^2^ (6, *N* = 154) = 16, *P* = 0.01										
20–40 years	61	40	10	16	38	62	12	20	1	2
40–60 years	80	52	5	6	33	41	37	46	5	6
60+ years	13	8	1	8	5	38	6	46	1	8
Sun protection by skin type: χ^2^ (18, *N* = 154) = 38, *P* = 0.004										
I	12	8	1	8	9	75	2	17	0	0
II	68	44	6	9	31	46	29	43	2	3
III	30	19	6	20	17	57	6	20	1	3
IV	19	12	1	5	9	47	7	37	2	11
V	9	6	0	0	0	0	8	89	1	11
VI	3	2	1	33	0	0	1	33	1	33
Unsure	13	8	1	8	10	77	2	15	0	0
Sun protection by SPF vehicle:χ^2^ (18, *N* = 236) = 82, *P* = 0.00										
Cream	46	19	8	17	22	48	16	35	0	0
Lotion	13	6	2	15	7	54	4	31	0	0
Spray	15	6	4	27	7	47	4	27	0	0
SPF cosmetic	26	11	6	23	17	65	3	12	0	0
Sunscreen	89	38	11	12	31	35	47	53	0	0
SPF cosmetic + sunscreen	13	6	4	31	8	62	1	8	0	0
Vehicle not specified	34	14	3	9	23	68	1	3	7	21
Sun protection by SPF brand: χ^2^ (30, *N* = 186) = 64, *P* < 0.001										
Altruist	37	20	5	14	13	35	14	38	5	14
Avène	6	3	1	17	3	50	1	17	1	17
CeraVe	9	5	2	22	3	33	2	22	2	22
Cetaphil	2	1	1	50	0	0	0	0	1	50
Eucerin	15	8	2	13	7	47	6	40	0	0
Garnier	7	4	1	14	4	57	2	29	0	0
La Roche-Posay	56	30	6	11	33	59	16	29	1	2
L’Oréal	3	2	0	0	2	67	1	33	0	0
Nivea	7	4	1	14	4	57	2	29	0	0
Own brand	8	4	1	13	5	63	2	25	0	0
Other	33	18	3	9	13	39	16	48	1	3
None	3	2	0	0	0	0	0	0	3	100
Sun protection by topical treatment use: χ^2^ (24, *N* = 193) = 57, *P* < 0.001										
Azelaic acid	20	10	4	20	13	65	3	15	0	0
AHAs/BHAs	2	1	0	0	2	100	0	0	0	0
Ivermectin	2	1	0	0	0	0	1	50	1	50
Nicotinamide	28	15	5	18	19	68	4	14	0	0
Tretinoin	32	17	4	13	19	59	8	25	1	3
Retinal	5	3	0	0	3	60	2	40	0	0
Retinol	35	18	3	9	26	74	6	17	0	0
Vitamin C	7	4	1	14	5	71	1	14	0	0
None	62	32	5	8	18	29	34	55	5	8
Sun protection by facial skin disease: χ^2^ (21, *N* = 177) = 25, *P* = 0.25										
Acne	23	13	4	17	14	61	5	22	0	0
Actinic field change	10	6	2	20	3	30	5	50	0	0
Eczema	10	6	3	30	2	20	5	50	0	0
Melasma	11	6	3	27	5	45	3	27	0	0
Psoriasis	2	1	0	0	0	0	2	100	0	0
Rosacea	16	9	1	6	8	50	6	38	1	6
Seborrhoeic dermatitis	12	7	0	0	7	58	5	42	0	0
None	93	53	6	6	46	49	35	38	6	6

AHAs/BHAs, alpha-hydroxy acids/beta-hydroxy acids; GPwER, general practitioner with an extended role; SPF, sun protection factor.

Among Fitzpatrick skin types, type VI had the highest proportion of clinicians applying SPF more than once daily (33%, *n* = 1/3), although this group was under-represented. Type III had the second highest frequency of multiple SPF applications (20%, *n* = 6/30), followed by type II (9%, *n* = 6/68), type I (8%, *n* = 1/12), type IV (5%, *n* = 1/19) and type V (0%, *n* = 0/9). Clinicians with Fitzpatrick skin type I were the most likely to apply SPF once daily (75%, *n* = 9/12), while clinicians with skin type V were most likely to use SPF only when they exposed their skin to the sun (89%, *n* = 8/9), followed by those with type II skin (43%, *n* = 29/68). The group of clinicians with type VI skin had the highest proportion who did not use SPF daily (33%, *n* = 1/3).

Clinicians using a cosmetic containing SPF and a standalone SPF were the most likely to apply SPF more than once daily (31%), followed by those using spray vehicles (27%). In contrast, 65% of clinicians relying solely on SPF-containing cosmetics applied SPF-containing just once daily.

Among specific SPF brands, the highest rates of SPF application more than once daily were observed among Cetaphil users (50%), followed by Avène users (17%), and users of Altruist, Garnier and Nivea products (14% each). Conversely, users of L’Oréal had the highest proportion of single daily SPF applications (67%), followed by own-brand users (63%) and La Roche-Posay product users (59%). Respondents were allowed to select multiple brands, and brands categorized as ‘other’ were represented by a single user each.

Regarding topical treatments, azelaic acid use was most associated with applying SPF more than once daily (20%), followed by nicotinamide (18%), vitamin C (14%) and tretinoin (13%). No significant relationship was identified between facial skin disease and SPF application frequency.

Training grade, age, sex, skin type, SPF brand/vehicle preference and topical treatment use significantly influenced SPF application frequency, as shown by χ^2^ testing. These factors may also affect clinicians’ frequency of sun protection counselling and patient adherence to recommendations.

Optimal SPF use was most common among those combining SPF-containing cosmetics with sunscreen (31%) and spray-on SPF users (27%). People with lighter skin types applied SPF more frequently; however, individuals with darker skin types may remain at risk due to delayed detection. Despite the increased sun sensitivity associated with tretinoin use, its users did not exhibit optimal SPF application, highlighting a need for stronger patient education in this area.

SPF brands associated with optimal use were typically available in mainstream pharmacies, underscoring the importance of accessibility. Female clinicians demonstrated better sun protection habits than their male counterparts, suggesting that male patients may benefit from targeted counselling. Notably, only 10.4% of clinicians (*n* = 16/154) reported applying SPF more than once daily, the recommended standard.

While these findings provide valuable insights for improving patient counselling, the results are not fully representative of all UK dermatologists. Some groups, such as those with Fitzpatrick V and VI skin types, were under-­represented. Response bias was minimized using multiple-choice and free-text survey options, and recall bias was reduced by focusing on daily SPF application habits.

This study identifies independent variables influencing SPF application frequency; however, the effects of these variables were not controlled for. Future research could further explore these determinants by controlling for confounders and investigating other factors, such as SPF ratings, sunscreen clothing and body-wide sunscreen use.

The significant variations in SPF application frequency among dermatology clinicians highlight the need for clearer clinical guidance. Only a minority of clinicians meet the recommended standard of reapplying SPF multiple times daily, indicating a potential area for improvement in professional practices.

Clinicians should emphasize the benefits of diligent sunscreen use, including protection against sun damage and its reversal.^10^ Concerns about sunscreen contributing to vitamin D deficiency, perpetuated by media narratives, have been refuted. Patients should be advised to apply SPF multiple times daily, year-round, and to supplement vitamin D during months of insufficient sun exposure.

Additionally, patients using SPF-containing cosmetics should be informed that a single application in the morning does not provide all-day protection^9^. Promoting practical options, such as spray-on SPFs, may enhance adherence to these recommendations. Dermatologists play a critical role in educating patients about effective sun protection, addressing gaps in understanding and fostering healthier behaviours.

## Data Availability

The data generated and analysed during this study are not publicly available at this time, as they will be used for a subsequent follow-up paper. Requests for access to the data may be considered on a case-by-case basis. Researchers interested in obtaining the data may contact the corresponding author with a detailed request, and access will be granted in accordance with applicable institutional and ethical guidelines.
